# Clopidogrel use After Myocardial Revascularization: Prevalence, Predictors, and One-Year Survival Rate

**DOI:** 10.5935/1678-9741.20160019

**Published:** 2016

**Authors:** Paulo Roberto L. Prates, Judson B. Williams, Rajendra H. Mehta, Susanna R. Stevens, Laine Thomas, Peter K. Smith, L. Kristin Newby, Renato A. K. Kalil, John H. Alexander, Renato D. Lopes

**Affiliations:** 1 Department of Cardiovascular Surgery, Instituto de Cardiologia-Fundação Universitária de Cardiologia, Porto Alegre, RS, Brazil.; 2 Clinical Research Center- Instituto de Cardiologia-Fundação Universitária de Cardiologia, Porto Alegre, RS, Brazil.; 3 Department of Surgery, Division of Cardiovascular and Thoracic Surgery, Duke University Medical Center, Durham, NC.; 4 Department of Medicine, Division of Cardiology, Duke University Medical Center, Durham, NC.; 5 Duke Clinical Research Institute, Durham, NC.

**Keywords:** Myocardial Revascularization, Coronary Artery Bypass, Blood Platelets

## Abstract

**Introduction:**

Antiplatelet therapy after coronary artery bypass graft (CABG) has been used. Little is known about the predictors and efficacy of clopidogrel in this scenario.

**Objective:**

Identify predictors of clopidogrel following CABG.

**Methods:**

We evaluated 5404 patients who underwent CABG between 2000 and 2009 at Duke University Medical Center. We excluded patients undergoing concomitant valve surgery, those who had postoperative bleeding or death before discharge. Postoperative clopidogrel was left to the discretion of the attending physician. Adjusted risk for 1-year mortality was compared between patients receiving and not receiving clopidogrel during hospitalization after undergoing CABG.

**Results:**

At hospital discharge, 931 (17.2%) patients were receiving clopidogrel. Comparing patients not receiving clopidogrel at discharge, users had more comorbidities, including hyperlipidemia, hypertension, heart failure, peripheral arterial disease and cerebrovascular disease. Patients who received aspirin during hospitalization were less likely to receive clopidogrel at discharge (*P*≤0.0001). Clopidogrel was associated with similar 1-year mortality compared with those who did not use clopidogrel (4.4% *vs.* 4.5%, *P*=0.72). There was, however, an interaction between the use of cardiopulmonary bypass and clopidogrel, with lower 1-year mortality in patients undergoing off-pump CABG who received clopidogrel, but not those undergoing conventional CABG (2.6% *vs* 5.6%, *P* Interaction = 0.032).

**Conclusion:**

Clopidogrel was used in nearly one-fifth of patients after CABG. Its use was not associated with lower mortality after 1 year in general, but lower mortality rate in those undergoing off-pump CABG. Randomized clinical trials are needed to determine the benefit of routine use of clopidogrel in CABG.

**Table:** 

Abbreviations, acronyms & symbols	
CABG	= Coronary artery bypass graft
CI	= Confidence interval
CURE	= Clopidogrel in unstable angina to prevent recurrent ischemic Events
HR	= Hazard risk
PCI	= Percutaneous coronary intervention
PREVENT IV	= Project of Ex-vivo Vein graft ENgineering via Transfection IV

## INTRODUCTION

Antiplatelet therapy is beneficial in secondary prevention following coronary artery bypass graft (CABG) surgery; however, there is no consensus about when to initiate therapy, how to dose, or the optimal combination of agents^[1,4]^. Clopidogrel also reduces ischemic events and mortality in patients with coronary and peripheral arterial disease^[5,8]^. While the use of aspirin after CABG surgery is widespread^[2,4,9,10]^, clopidogrel, the most commonly used P2Y_12_ inhibitor, in addition to aspirin, has been used less often than aspirin alone^[11]^.

Although it has been shown that antiplatelet therapy improves the patency of venous grafts^[2,4,12]^, little is known about which patients are receiving clopidogrel after CABG surgery or about its association with patient outcomes. In this large singlecenter study we described the clinical and surgical characteristics and predictors of patients receiving clopidogrel following CABG surgery, determined the rates of clopidogrel use after 1 year, and compared the 1-year risk-adjusted mortality in patients receiving and not receiving clopidogrel.

## METHODS

### Study Patients

We evaluated 6588 patients undergoing CABG surgery between 2000 and 2007 at Duke University Medical Center (Durham, NC, USA). A total of 815 patients were excluded for concomitant valve surgery, 177 were excluded for in-hospital reoperation for bleeding or anticoagulation complications, 188 died prior to discharge, and 4 additional patients who had undergone CABG surgery within 9 days were excluded, resulting in a final sample size of 5404. Patients undergoing urgent or elective procedures were included. This study was approved by the Duke University Health System Institutional Review Board. The requirement for individual consent was waived. All patients undergoing surgical procedures signed an informed consent form according to the data collected during hospitalization which can be used in research.

### Surgical Procedures

On- and off-pump CABG procedures were performed during the study period, including both urgent and elective procedures. All patients were operated by the same group of surgeons that consists of 19 professionals. After median sternotomy, patients underwent conventional CABG surgery with the use of internal mammary arteries whenever possible. When performed on pump CABG, standard cardiopulmonary bypass was used, typically with both anterograde and retrograde cold blood cardioplegia.

### Clinical Follow-Up

Discharge clopidogrel use was determined by in-hospital medication records, that was found in the electronic medical record, and was defined as administration at any time the day after surgery through the date of discharge. The files were accessed by the institution's research group. In addition to clinical data collected during patient visits, medication use and survival were determined using self-administered mailed questionnaires and telephone follow-up for those who did not return questionnaires. Deaths reported by Duke hospitals, the National Death Index, and the Social Security Death Index were used to confirm or supplement the follow-up surveys for mortality information. These actions were also performed by the institution's research group. Operative mortality was defined as death occurring within 30 days of the index procedure or before discharge.

### Statistical Analyses

Summary statistics were expressed as medians (25^th^, 75^th^ percentiles) for continuous variables and numbers (percentage) for categorical variables. Baseline and in-hospital patient characteristics for those who did and did not receive in-hospital clopidogrel following CABG surgery were compared using the Wilcoxon rank sum test for continuous variables and chisquare or Fisher's exact tests for categorical variables. Because of recent findings demonstrating differences between patients undergoing on- and off-pump CABG surgery^[13]^, unadjusted Kaplan-Meier rates for 1-year survival are shown for the 4-level stratification of patients who did and did not receive clopidogrel and had on-pump *versus* off-pump CABG surgery.

Multivariable logistic regression was used to determine the association between baseline and in-hospital demographics and clinical factors with in-hospital clopidogrel use following CABG surgery. All variables as well as their interactions with on- and off-pump CABG surgery were considered for inclusion using backward elimination with a requirement of alpha <0.05 for retention (Tables 1 and 2).

**Table 1:** Baseline characteristics according to clopidogrel use after CABG.

Variable	All Patients (N=5404)	No Clopidogrel at Discharge (N=4473)	Clopidogrel at Discharge (N=931)	*P* Value
Age, median (25^th^, 75^th^), yrs*	64 (56.72)	64 (56.72)	63 (55.72)	0.03
Female sex, no. (%) *	1593 (29.5)	1276 (28.5)	317 (34.0)	<0.001
White race, no. (%)	4130 (77.5)	3413 (77.4)	717 (78.4)	0.51
Weight, median (25^th^, 75^th^), kg	84 (73.97)	85 (74.97)	83 (73.96)	0.13
Medical history, no. (%)				
Hypertension	4272 (79.1)	3499 (78.2)	773 (83.0)	0.001
Diabetes mellitus *	1984 (36.7)	1632 (36.5)	352 (37.8)	0.45
Smoking status				
Current	1485 (29.5)	1242 (29.9)	243 (27.5)	
Former	1431 (28.4)	1127 (27.2)	304 (34.4)	<0.001
Never	2115 (42.0)	1779 (42.9)	336 (38.1)	
Hyperlipidemia	3843 (71.1)	3136 (70.1)	707 (75.9)	<0.001
Chronic lung disease *	611 (11.3)	507 (11.3)	104 (11.2)	0.89
Any prior MI *	2738 (50.7)	2252 (50.3)	486 (52.2)	0.30
Recent MI (30 days)	1670 (34.2)	1397 (34.5)	273 (33.1)	0.43
Prior PCI	838 (15.5)	663 (14.8)	175 (18.8)	0.002
Prior CABG	238 (4.4)	177 (4.0)	61 (6.6)	<0.001
Prior valve procedure	36 (0.7)	30 (0.7)	6 (0.6)	0.93
CHF within prior 2 weeks *	865 (16.0)	743 (16.6)	122 (13.1)	0.008
History of cerebrovascular disease *	720 (13.3)	576 (12.9)	144 (15.5)	0.03
History of peripheral vascular disease *	900 (16.7)	724 (16.2)	176 (18.9)	0.04
Patient currently on dialysis	111 (2.1)	95 (2.1)	16 (1.7)	0.43
Renal failure *	56 (1.0)	48 (1.1)	8 (0.9)	0.56
Cardiogenic shock during the procedure	15 (0.3)	15 (0.3)	0 (0)	0.09
Presenting features				
Creatinine (most recent in past 60 days), median (25^th^, 75^th^) *	1.0 (0.9, 1.2)	1.0 (0.9, 1.2)	1.0 (0.9 - 1.2)	0.35
GFR (most recent in past 60 days), median (25^th^, 75^th^) *	77 (63, 92)	77 (63, 91)	78 (63 - 93)	0.24
EF (most recent in past 60 days), median (25^th^, ^75th^)*	51 (40, 71)	51 (40, 68)	51 (43 - 75)	0.002
Number of vessels ≥ 50% stenosed, no. (%)				
0	19 (0.4)	17 (0.4)	2 (0.2)	
1	296 (5.5)	215 (4.8)	81 (8.7)	<0.001
2	964 (17.8)	783 (17.5)	181 (19.4)	
3	4125 (76.3)	3458 (77.3)	667 (71.6)	
Left main disease > 50%, no. (%)	1534 (28.4)	1252 (28.0)	282 (30.3)	0.16
Preoperative antiplatelet agents, no. (%)				
Aspirin administered prior to CABG	3426 (63.4)	2844 (63.6)	582 (62.5)	0.54
Clopidogrel administered prior to CABG	400 (7.4)	297 (6.6)	103 (11.1)	<0.001

* Included in multivariable model for mortality.

CABG=coronary artery bypass grafting; CHF=congestive heart failure; EF=ejection fraction; GFR=glomerular filtration rate; MI=myocardial infarction; PCI=percutaneous coronary intervention

**Table 2:** Operative and postoperative characteristics according to clopidogrel use after CABG.

Variable	All Patients (N=5404)	No Clopidogrel at Discharge (N=4473)	Clopidogrel at Discharge (N=931)	*P* Value
Surgery type, no. (%)				
Elective	1487 (27.5)	1306 (29.2)	181 (19.4)	
Emergent	299 (5.5)	246 (5.5)	53 (5.7)	<0.001
Urgent	3618 (67.0)	2921 (65.3)	697 (74.9)	
Left or right IMA, no. (%)*	4940 (91.4)	4141 (92.6)	799 (85.8)	<0.001
SVG harvested endoscopically, no. (%)*	4948 (98.0)	4129 (98.1)	819 (97.7)	0.48
On-pump surgery, no. (%)*	4614 (85.4)	4061 (90.8)	553 (59.4)	<0.001
Cross-clamp time, median (25^th^, 75^th^)*	60 (39, 80)	61 (43, 80)	44 (0, 78)	<0.001
Perfusion time, median (25^th^, 75^th^)*	109 (83, 135)	110 (88, 134)	94 (0, 135)	<0.001
Number of grafts, no. (%)				
1	280 (5.2)	199 (4.4)	81 (8.7)	
2	820 (15.2)	636 (14.2)	184 (19.8)	<0.001
3	2363 (43.7)	1982 (44.3)	381 (40.9)	
≥4	1941 (35.9)	1656 (37.0)	285 (30.6)	
Worst target artery quality, no. (%)*				
Good	2094 (39.2)	1843 (41.6)	251 (27.7)	
Fair	2298 (43.0)	1898 (42.8)	400 (44.2)	<0.001
Poor	946 (17.7)	692 (15.6)	254 (28.1)	
Worst graft quality, no. (%)*				
Good	3916 (73.6)	3359 (76.0)	557 (61.6)	
Fair	1206 (22.7)	911 (20.6)	295 (32.6)	<0.001
Poor	202 (3.8)	150 (3.4)	52 (5.8)	
Type of graft, no. (%)				
Left saphenous vein	4716 (87.3)	3953 (88.4)	763 (82.0)	<0.001
Right saphenous vein	1161 (21.5)	907 (20.3)	254 (27.3)	<0.001
Both left & right saphenous veins	785 (14.5)	614 (13.7)	171 (18.4)	<0.001
Left internal thoracic artery	4875 (90.2)	4090 (91.4)	785 (84.4)	<0.001
Right internal thoracic artery	188 (3.5)	156 (3.5)	32 (3.4)	0.94
Both left & right internal thoracic arteries	123 (2.3)	104 (2.3)	19 (2.0)	0.60
Left radial artery	305 (5.6)	201 (4.5)	104 (11.2)	<0.001
Right radial artery	50 (0.9)	37 (0.8)	13 (1.4)	0.10
Both left & right radial arteries	17 (0.3)	12 (0.3)	5 (0.5)	0.20
Length of stay, median (25^th^, 75^th^)*	9 (7, 12)	9 (7, 12)	9 (7, 12)	0.67
Patient discharged on warfarin, no. (%)*	208 (3.8)	178 (4.0)	30 (3.2)	0.27
Aspirin after surgery and before discharge, no. (%)*	5303 (98.1)	4403 (98.4)	900 (96.7)	<0.001
MI occurs after surgery and before discharge, no. (%)*	13 (0.2)	5 (0.1)	8 (0.9)	<0.001
Cerebrovascular accident after surgery and before discharge, no. (%)*	112 (2.1)	91 (2.0)	21 (2.3)	0.67
Atrial fibrillation before discharge, no. (%)*	930 (17.2)	794 (17.8)	136 (14.6)	0.02

* Included in multivariable model for mortality.

IMA=internal mammary artery; MI=myocardial infarction; SVG=saphenous vein graft

Cox proportional hazards analysis was performed to evaluate the association between post-CABG clopidogrel use and 1-year mortality. We adjusted for covariates identified in the PRoject of Ex-vivo Vein graft ENgineering via Transfection IV (PREVENT IV) trial mortality model^[13]^, developed in a similar CABG population, and included the most closely related variables available in our dataset. In addition, we also adjusted for aspirin use after CABG surgery, warfarin use at discharge, on- and off-pump CABG-surgery, in-hospital cerebrovascular accident and renal failure. Adjusted survival curves are shown for the clopidogrel effect in the multivariable Cox mortality model. The model was repeated with the inclusion of the interaction of clopidogrel with on- and off-pump CABG surgery; adjusted survival curves for this interaction are shown.

For the multivariable logistic and Cox models, continuous and ordinal variables were tested for linearity over the log hazard and were transformed as necessary to meet this modeling assumption. The proportional hazards assumption was checked for each variable in the mortality model and there were no deviations of concern. Statistical analyses were performed using SAS version 9.1 (SAS Institute, Inc., Cary, NC, USA).

## RESULTS

### Study Population and Baseline Characteristics

Total of 5404 patients who underwent CABG surgery from 2000-2007 where evaluated. Among these patients, 931 (17.2%) received clopidogrel after CABG surgery. Patients who received clopidogrel, 789 were alive with complete medication information after 1 year and 314 (39.8%) were still taking clopidogrel. Among the patients not taking clopidogrel after surgery, 8.9% (345/3868) were taking it after 1 year. One-year mortality was related to the use of clopidogrel at any time in hospital postoperative evolution. The above data add information about using this medication after discharge.

Patient baseline characteristics are shown in Table 1. When compared with patients who did not receive clopidogrel, those who did were younger and had more comorbidities including hyperlipidemia, hypertension, peripheral arterial disease, and cerebrovascular disease but less heart failure in the prior 2 weeks. They were also more likely to have undergone prior percutaneous coronary intervention (PCI) or CABG surgery. Post-CABG clopidogrel users were also more likely to have received clopidogrel in the preoperative period. Overall, aspirin was used in 98.1% of patients after CABG surgery (96.7% with and 98.4% without clopidogrel after CABG surgery).

### Surgical Characteristics

The main surgical procedure characteristics are shown in Table 2. Patients who did not receive clopidogrel more often underwent elective surgery (29.2% *vs.* 19.4%) while those receiving clopidogrel more often underwent urgent procedures (65.3% *vs.* 74.9%). Clopidogrel users were more likely to have bad quality grafts than patients who did not receive clopidogrel. The hospital length of stay was similar among the 2 groups.

In the overall population, 4716 (87.3%) patients had left saphenous vein grafts harvested, 1161 (21.5%) had right saphenous vein grafts harvested, and 785 (14.5%) had saphenous vein grafts from both left and right legs harvested (Table 2). A total of 4875 (90.2%) patients had a left internal thoracic artery graft, 188 (3.5%) had a right internal thoracic artery graft, and 123 (2.3%) had both internal thoracic artery grafts. Left radial grafts were used in 305 (5.6%) patients, 50 (0.9%) patients had a right radial artery graft, and 17 (0.3%) had both a right and left radial artery graft. While right saphenous vein grafts and left radial artery grafts were more commonly used in patients discharged with clopidogrel, left saphenous vein grafts and left internal thoracic artery grafts were more often used in patients discharged without clopidogrel (Table 2).

### Predictors of Clopidogrel Use

The predictors of clopidogrel use are shown in Table 3. Patients who had worse target artery or graft quality, left main disease, prolonged perfusion time, clopidogrel before surgery, or prior PCI were more likely to receive clopidogrel after CABG surgery. Advanced age, internal mammary artery graft, elective surgery, and aspirin use before surgery or at discharge were associated with a lower probability of clopidogrel use following CABG surgery.

**Table 3:** Multivariable associations with clopidogrel use after CABG (N=4887).

Variable	Chi-square	OR (95% CI)	*P* value
Worst target artery quality, OR for 1 category increase	671,789	1.70 (1.50, 1.94)	<0.0001
Left or right IMA	197,168	0.55 (0.42, 0.72)	<0.0001
Worst graft quality, OR for 1 category increase	160,361	1.36 (1.17, 1.58)	<0.0001
Age, OR for 10-year increase	134,104	0.86 (0.79, 0.93)	0.0003
Left main disease	127,628	1.40 (1.16, 1.69)	0.0004
MI before discharge	115,781	10.85 (2.75, 42.82)	0.0007
Surgery type (reference is elective)			
Emergency	127,735	1.33 (0.88, 1.99)	0.0017
Urgent		1.52 (1.21, 1.92)	
Ejection fraction, OR for 10% increase			
Linear spline ≥67, OR for off pump	98,291	4.10 (2.48, 6.77)	0.0017*
Linear spline ≥67, OR for on pump		1.75 (1.23, 2.49)	
Linear spline ≤67	47,879	0.92 (0.85, 0.99)	0.0287
Number of grafts, OR for off pump	88,668	1.08 (0.88, 1.32)	0.0029*
Number of grafts, OR for on pump		0.75 (0.66, 0.86)	
Aspirin at discharge	83,606	0.46 (0.27, 0.78)	0.0038
Pre-CABG clopidogrel	76,374	1.52 (1.13, 2.04)	0.0057
Perfusion time, OR for 30-minute increase	74,737	1.13 (1.04, 1.24)	0.0063
SVG harvested endoscopically, OR for off pump	73,470	4.66 (1.33, 16.33)	0.0067*
SVG harvested endoscopically, OR for on pump		0.67 (0.36, 1.26)	
Congestive heart failure	72,702	0.71 (0.55, 0.91)	0.0070
Pre-CABG aspirin	69,593	0.77 (0.63, 0.93)	0.0083
History of hypertension	58,004	1.32 (1.05, 1.65)	0.0160
History of PCI	55,437	1.31 (1.05, 1.63)	0.0185
Cross-clamp time, OR for 30-minute increase	53,599	1.18 (1.03, 1.35)	0.0206
Cerebrovascular accident before discharge, OR for off pump	51,980	0.24 (0.05, 1.16)	0.0226*
Cerebrovascular accident before discharge, OR for on pump		1.68 (0.96, 2.94)	
Atrial fibrillation before discharge	51,469	0.76 (0.59, 0.96)	0.0233
Discharge warfarin, OR for off pump	40,785	1.63 (0.60, 4.43)	0.0434*
Discharge warfarin, OR for on pump		0.50 (0.28, 0.90)	
History of cerebrovascular disease	40,576	1.28 (1.01, 1.62)	0.0440

* *P* value for interaction term of variable with on/off-pump.

CI=confidence interval; IMA=internal mammary artery; MI=myocardial infarction; OR=odds ratio; PCI=percutaneous coronary intervention; SVG=saphenous vein graft

### 1-Year Mortality

**Fig. 1:**
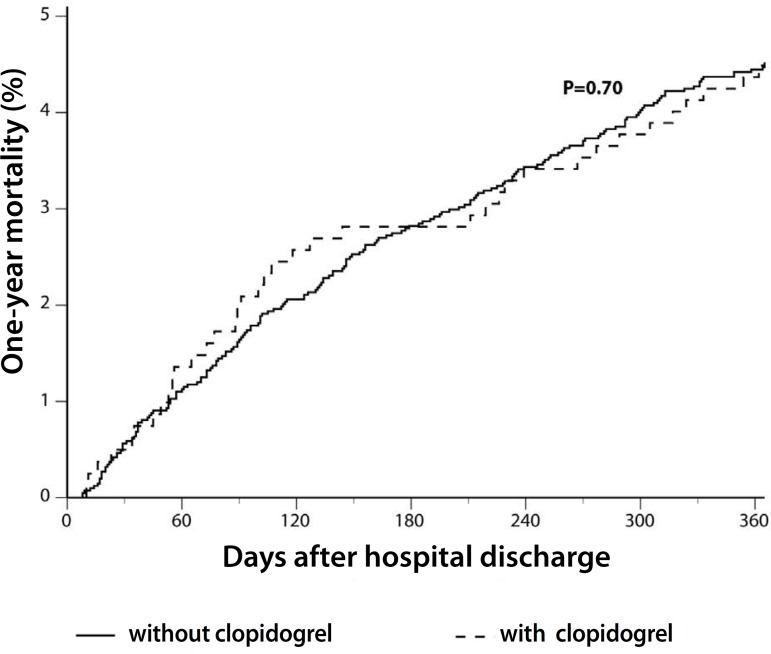
One-year mortality according to the clopidrogel use after CABG.

**Fig. 2:**
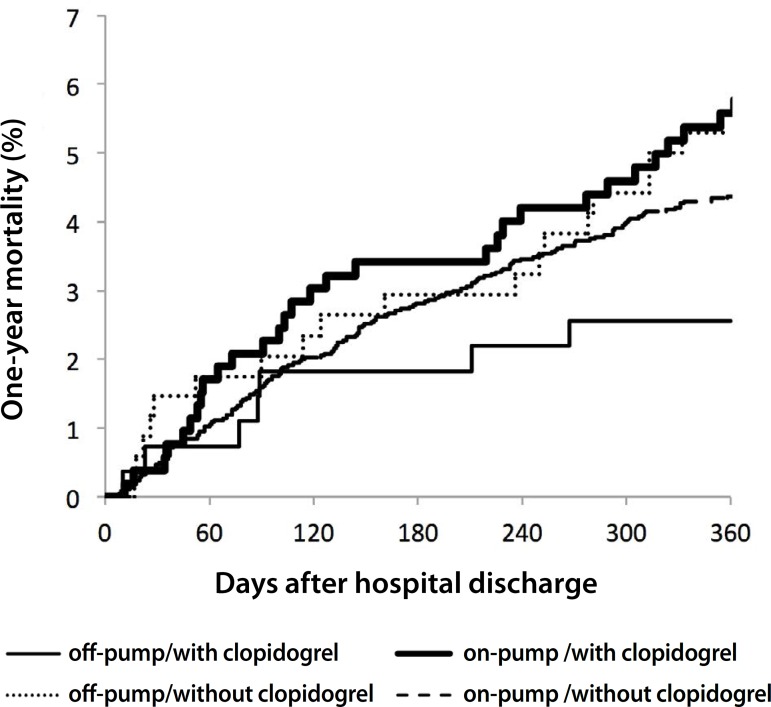
One-year mortality according to the clopidrogel use after on-pump and off-pump CABG.

Clopidogrel use was associated with similar 1-year mortality (4.7% *vs.* 4.5%, adjusted hazard ratio (HR) 1.08, 95% confidence interval (CI) 0.73-1.59; *P*=0.70) compared with those not using clopidogrel (Table 4, Figure 1). However, there was an interaction between use of cardiopulmonary bypass and clopidogrel, with lower 1-year mortality with clopidogrel in patients undergoing off-pump CABG surgery (adjusted HR 0.47, 95% CI 0.19-1.13), but not in those undergoing on-pump CABG surgery (adjusted HR 1.35, 95% CI 0.89-2.05; *P* interaction=0.032) (Figure 2).

**Table 4:** Kaplan-Meier rates for 1-year mortality according clopidogrel use *versus* not stratified by off and on pump surgeries.

	Alive	Death	Total
Off pump			
No clopidogrel, no. (%)	392 (95.1)	20 (4.9)	412
Clopidogrel, no. (%)	368 (97.4)	10 (2.6)	378
Total	760	30	790
On pump*			
No clopidogrel, no. (%)	3877* (95.5)	183 (4.5)	4060
Clopidogrel, no. (%)	521* (94.4)	31 (5.6)	552
Total	4398	214	4612

* One on-pump patient without and one on-pump patient with clopidogrel after CABG were censored before the 1-year follow-up period. These patients are not included in the alive counts after 1 year but are reflected in the Kaplan-Meier rate.

## DISCUSSION

Our study has 2 main findings. First, at Duke University Medical Center, postoperative clopidogrel is used in almost one-fifth of the patients undergoing CABG surgery. These patients tend to be sicker and have more comorbidities than those who do not receive clopidogrel after surgery. We also identified several key factors associated with clopidogrel use after CABG surgery. Second, clopidogrel use was associated with similar 1-year mortality compared with those patients not using it. However, there was an interaction between the use of cardiopulmonary bypass and clopidogrel, with lower 1-year mortality with clopidogrel among patients undergoing off-pump CABG surgery and higher 1-year mortality with clopidogrel among those undergoing on-pump CABG surgery.

Previous studies have demonstrated the benefit of antiplatelet agents, particularly aspirin, after CABG surgery, although there is no consensus on when to initiate and what dose should be used. Moreover, these studies do not directly show the impact of therapy on mid- and long-term mortality^[3,4,7]^. There are published reports of controlled trials showing benefit of different antiplatelet therapies, including aspirin alone^[9,14]^, aspirin plus dipyridamole^[14,16]^, and aspirin plus sulfinpyrazone^[17]^. More recent studies have demonstrated improved graft patency with the use of clopidogrel^[7,12]^. While the success of the surgical procedure is most critical to the patency of a graft, understanding the relationship between clopidogrel use and mortality and other hard clinical outcomes is critical.

Clopidogrel use in patients with acute coronary syndromes demonstrates benefit^[5,6,18]^. Treatment with clopidogrel reduced the risk of myocardial infarction and recurrent ischemia, with a trend toward lower rates of cerebrovascular accident and death from cardiovascular causes^[5,6,19]^. Antiplatelet therapy with aspirin has led to improvements in vein graft patency when started early after CABG^[4,7,20]^. The combination of clopidogrel and aspirin after off-pump CABG surgery was previously suggested to reduce cardiac events and mortality^[12]^ as well as improve graft patency in a single-center trial of 249 patients (91.6% for aspirin plus clopidogrel *vs.* 85.7% for aspirin alone; *P*=0.043) ^[7]^.

While it remains unknown why some patients received clopidogrel and others did not, our study identified several factors associated with clopidogrel use following CABG surgery: target vessel quality, graft quality, age, congestive heart failure, cerebrovascular accident, prior myocardial infarction, prior PCI, prior CABG surgery, and aspirin use at hospital discharge. The strongest predictor of clopidogrel use after CABG surgery was worse target artery quality. Importantly, almost every patient (98.1%) received aspirin after the CABG surgery and this was significantly associated with less use of clopidogrel during the hospital stay. Whether the patients were treated on or off pump, it appears that clopidogrel was generally chosen for younger patients (perhaps balancing bleeding risks) with poor target artery quality, cerebrovascular disease, and previous coronary interventions. Without randomized data on clopidogrel use following CABG surgery, our study provides insights about potential factors associated with its use that might help physicians decide when to use clopidogrel in this clinical setting.

In a subgroup analysis from the PREVENT IV study, in which all patients received aspirin at hospital discharge, clopidogrel use was associated with a trend for higher rates of occluded vein grafts during 12-18 months (49% *vs.* 39%; adjusted odds ratio 1.26; *P*=0.08) and with similar composite rates of death, myocardial infarction, or revascularization (27% *vs.* 24%; adjusted HR 1.10; *P*=0.38) in 5 years compared with those without it^[13]^. This study found a significant interaction between use of cardiopulmonary bypass and clopidogrel. Similarly, in our study, the administration of clopidogrel during the hospital stay was not associated with overall 1-year mortality in patients undergoing CABG surgery, even in those cases where an emergency or urgent surgery was needed. In patients undergoing off-pump surgery, we found that clopidogrel use was associated with higher 1-year survival; however, in patients undergoing on-pump surgery, clopidogrel use was associated with higher 1-year mortality.

Studies show different conclusions regarding the results found when the techniques compared with and without cardiopulmonary bypass^[21,23]^. On the other hand, several investigators have indicated that off-pump CABG surgery may increase the risk of thrombosis due to augmented thrombotic activity^[24,25]^. There is a well-known phenomenon of thrombotic activity following major general surgery and it is expected after major procedures^[25]^. In fact, Mariani et al.^[25]^ demonstrated that thrombotic activity is increased in the first 24 hours after off-pump surgery. Clopidogrel appeared to have a role in decreasing clotting and protecting the patency of anastomoses. In on-pump surgery, there is a well described decrease in platelet function that could bring benefits for graft patency^[26,27]^. This benefit does not occur in off-pump surgery where platelet function tends to be closer to normal, leaving more room for benefit of an antiplatelet agent such as clopidogrel. It is known that extracorporeal circulation leads to a decrease in blood coagulation activity, mainly due to consumption of factors and reduction of platelet activity^[26]^. This could have a protective impact on patients undergoing on-pump CABG surgery. In this situation, the pharmacological activity of clopidogrel may not have as much of a role and this may have contributed to our findings.

In the Clopidogrel in Unstable angina to prevent Recurrent ischemic Events (CURE) trial, patients randomized to clopidogrel in addition to aspirin had a 20% reduction in cardiovascular death, myocardial infarction, or cerebrovascular accident in the 9^th^ month in the follow-up period. Among patients who underwent CABG surgery, the apparent benefit of clopidogrel was tempered by a higher major bleeding rate among clopidogrel-treated patients (9.6% *vs.* 7.5%, respectively). Importantly, these patients were already on clopidogrel when undergoing CABG surgery and did not start clopidogrel for the first time after surgery. Based in large part on the CURE trial data, the current American College of Cardiology/American Heart Association/Society of Thoracic Surgeons guidelines recommend withholding therapy for 5 days among acute coronary syndrome patients requiring CABG surgery^[28]^.

At the present time, there are not adequate randomized clinical trial data to determine whether adding clopidogrel to aspirin prevents adverse clinical outcomes (death, myocardial infarction, cerebrovascular accident, unstable angina, or recurrence of angina) after CABG surgery. Despite this, clopidogrel is widely but inconsistently prescribed in patients after CABG surgery with stable coronary disease^[18]^, a practice supported primarily by subgroup analyses and observational data^[12,29,31]^. In our study, clopidogrel was used more often in patients with poor graft quality, which suggests that cardiologists and cardiac surgeons might be using clopidogrel after CABG surgery in patients with worse coronary disease. While also observational, the data we present do not support a beneficial effect of dual antiplatelet therapy following CABG surgery, although there may be some benefit in the off-pump setting.

### Limitations

Our study has several limitations to consider. First, this is an observational study and one cannot account for unmeasured confounders. Thus, a cause and effect relationship between clopidogrel use and mortality cannot be assessed. Second, this is a single-center study and caution should be taken when generalizing our results to other institutions or settings. Third, while target artery quality was measured, other intraoperative technical factors were not measured and may play a role in identifying candidates for dual antiplatelet therapy after CABG surgery. Fourth, discharge clopidogrel was determined using inhospital medication records and it was defined as administration at any time the day after surgery through the date of discharge. Therefore, the term "clopidogrel at discharge" is not consistent and does not necessarily means clopidogrel use at the time of discharge. In addition, we did not have information on 1-year medication use for all patients. We demonstrated that only around one-third of the patients who were discharged on clopidogrel were on it for 1 year, and less than 10% of patients who did not receive clopidogrel at discharge were on it for 1 year. Unfortunately, we did not collect the reasons for stopping and starting clopidogrel following CABG surgery. Nonetheless, this is one of the few studies that was able to describe medication use in 1 year, which provides important insights about adherence and medication persistence.

## CONCLUSION

At our institution, clopidogrel was used in almost one-fifth of patients following CABG surgery. Its use was not associated with improved overall 1-year survival, yet may have some benefit among those receiving off-pump CABG. Adequately powered randomized clinical trials are needed to determine whether there is a role for routine or selected use of clopidogrel or newer antiplatelet agents after CABG surgery.

**Table:** 

Authors' roles & responsibilities	
PRLP	Analysis and/or data interpretation; manuscript redaction or critical review of its content; final manuscript approval
JBW	Analysis and/or data interpretation; manuscript redaction or critical review of its content; final manuscript approval
RHM	Analysis and/or data interpretation; manuscript redaction or critical review of its content; final manuscript approval
SRS	Statistical analysis; manuscript redaction or critical review of its content; final manuscript approval
LT	Statistical analysis; manuscript redaction or critical review of its content; final manuscript approval
PKS	Analysis and/or data interpretation; manuscript redaction or critical review of its content; final manuscript approval
LKN	Analysis and/or data interpretation; manuscript redaction or critical review of its content; final manuscript approval
RAKK	Analysis and/or data interpretation; manuscript redaction or critical review of its content; final manuscript approval
JHA	Conception and design study; analysis and/or data interpretation; manuscript redaction or critical review of its content; final manuscript approval
RDL	Conception and design study; analysis and/or data interpretation; manuscript redaction or critical review of its content; final manuscript approval
